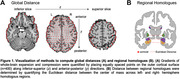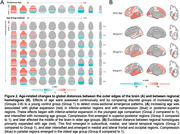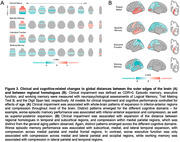# Compression and expansion in the aging brain is associated with clinical and cognitive outcomes

**DOI:** 10.1002/alz.093017

**Published:** 2025-01-09

**Authors:** Jenna N. Adams, Yuritza Y Escalante, Michael A. Yassa, Niels Janssen

**Affiliations:** ^1^ University of California, Irvine, Irvine, CA USA; ^2^ Universidad de La Laguna, Santa Cruz de Tenerife Spain

## Abstract

**Background:**

The brain undergoes structural changes during aging, such as gray matter loss, enlarged ventricles, and sulcal widening. However, previous studies have primarily investigated these changes in isolation, without describing the complex spatial relationships between overall brain shape and regions. Here, we tested how gradients of expansion and compression of the global shape of the brain as well as between homologous brain regions across hemispheres are affected by age, and whether these changes further contribute to clinical impairment and cognitive deficits in older adults.

**Method:**

We analyzed 2,039 structural MRIs from the Open Access Series of Imaging Studies (OASIS) dataset (42‐97 years; 56%F). MRIs were preprocessed using Freesurfer version v6.0. Gradients of whole‐brain expansion and compression were quantified by placing equally spaced points on the outer cortical surface (n=400) along inferior‐superior and anterior‐posterior directions (Figure 1A). To assess regional changes, we measured the Euclidean distance between left and right hemisphere homologues (Figure 1B). Age effects were evaluated continuously and by comparing groups of increasing age to a young control group (42.7‐59.9 years). Clinical impairment was defined as a Clinical Dementia Rating (CDR) >0, and neuropsychological testing were used to probe cognitive deficits.

**Result:**

Increasing age was associated with global expansion in inferior‐anterior regions and with compression in posterior‐superior regions (Figure 2A). Cross‐hemisphere distances between regional homologues showed a progressive pattern of expansion in subcortical and medial/lateral temporal lobes and compression in posterior parietal areas across increasing age groups (Figure 2B). Controlling for age, these patterns were exacerbated in clinically impaired older adults (CDR>0) and related to episodic memory and executive function scores in a regionally‐specific manner (Figure 3). Global and regional results replicated in an independent sample of older adults (Cambridge Centre for Ageing and Neuroscience dataset; N=564, 30‐88 years, 51%F).

**Conclusion:**

During aging, the overall shape of the brain undergoes patterns of expansion within inferior‐anterior and subcortical regions, and compression within superior‐posterior regions, that further contribute to clinical impairment and cognitive deficits. This suggests that changes to the complex spatial anatomy and geometry of the aging brain may impact its ability to function efficiently and lead to cognitive dysfunction in older adults.